# Can blended learning and the flipped classroom improve student learning and satisfaction in Saudi Arabia?

**DOI:** 10.5116/ijme.57a7.83d4

**Published:** 2016-09-04

**Authors:** Muhammad R. Sajid, Abrar F. Laheji, Fayha Abothenain, Yezan Salam, Dina AlJayar, Akef Obeidat

**Affiliations:** 1 Department of Pathology, College of Medicine, Alfaisal University, Riyadh, Saudi Arabia; 2 College of Medicine, Alfaisal University, Riyadh, Saudi Arabia; 3 Department of Medical Education, College of Medicine, Alfaisal University, Riyadh, Saudi Arabia

**Keywords:** Blended learning, flipped classroom, online learning, asynchronous discussion boards

## Abstract

**Objectives:**

To evaluate student academic performance and perception towards blended learning and flipped classrooms in comparison to traditional teaching.

**Methods:**

This study was conducted during the hematology block on year three students. Five lectures were delivered online only. Asynchronous discussion boards were created where students could interact with colleagues and instructors. A flipped classroom was introduced with application exercises. Summative assessment results were compared with previous year results as a historical control for statistical significance. Student feedback regarding their blended learning experience was collected.

**Results:**

A total of 127 responses were obtained. Approximately 22.8% students felt all lectures should be delivered through didactic lecturing, while almost 35% felt that 20% of total lectures should be given online. Students expressed satisfaction with blended learning as a new and effective learning approach. The majority of students reported blended learning was helpful for exam preparation and concept clarification. However, a comparison of grades did not show a statistically significant increase in the academic performance of students taught via the blended learning method.

**Conclusions:**

Learning experiences can be enriched by adopting a blended method of instruction at various stages of undergraduate and postgraduate education. Our results suggest that blended learning, a relatively new concept in Saudi Arabia, shows promising results with higher student satisfaction. Flipped classrooms replace passive lecturing with active student-centered learning that enhances critical thinking and application, including information retention.

## Introduction

Education systems worldwide have advanced exponentially coincident with modern technology. E-learning (electronic learning) complements learner abilities and performance, providing them with increased control over learning hours, pace, and methods via various software packages.^1^ A combination of two instruction modes, e-learning and didactic (face-to-face) teaching is termed blended learning (BL).^2^^,^^3^ This approach circumvents availability of time and place, enhancing both student and faculty productivity. The implementation of a BL system through varying instructional approaches in a student-centered manner can create a positive and collaborative learning atmosphere.^3^  A statistically significant improvement in student performance was reported following the inclusion of e-learning within a graduate level public health program, in addition to favorable and enthusiastic learner reviews.^2^ The application of a blended learning approach in a microbiology course showed academic performance comparable to traditional learning.^4^ Edginton and Holbrook reported that students were initially apprehensive about decreased instructor communication in the blended learning system, but such concerns were superseded by increased attention towards their own time management skills. Nevertheless, face-to-face interaction was still highly valued.^5^ A qualitative study from an instructor’s perspective reported that although teachers faced some technological challenges, they also felt that use of electronic methods improves students’ performance by providing them with increased opportunities.^6^ Asynchronous online discussion boards have also shown their utility in enhancing the experience of students in the context of blended learning.^7^

Flipped or inverted classrooms are a blended learning modality, whereby traditional in-class lectures and homework exercises are reversed.^8^ The learning material is made available to students prior to the class to study at their own time, while in-class time is spent in interactive teaching activities.^8^ These activities include, but are not limited to: case-based learning, team based learning, project-based learning, inquiry-based learning, and cooperative learning.^9^ Flipped classrooms reduce passivity, enabling students to become active learners through reasoning and concept application,^8^ also facilitating increased student interaction with peers and instructors. By keeping students engaged in-class, such instructional approaches decrease distraction, enhancing retention and application of acquired information.^8^

BL can be applied at various stages within the scope of medical education, from theoretical acquisition of basic sciences, to clinical education, clinical practice, and postgraduate studies. Scant scientific literature is available critically appraising the impact of such blended methodology on clinical education of healthcare students; thus, additional reliable research is required to establish solid evidence.^10^^,^^11^ Furthermore, there are very few studies investigating similar applications of BL systems in educational institutions in the Middle East. This is one of the first applications of BL and flipped classroom in an institute in the Kingdom of Saudi Arabia. The purpose of this study is to analyze the academic performance of students and their perceptions after the application of blended learning and compare them to didactic teaching.

### Methods

### Study design and participants

This is a descriptive analytical study. A non-randomized purposive sample was used. Participants were year three medical students (n=154). Out of the 127 responses obtained, 64 were female and 63 were male. The research proposal was submitted to Internal Review Board (IRB) of Alfaisal University for approval. The study was conducted after the approval of the ethics board was received. The anonymity of participants was preserved and ethical regulations were duly followed.

### Procedure

The College of Medicine follows a spiral problem-based, self-directed curriculum, in which patients and problems are studied from multiple stand-points. Students in years one and two study basic structure and function, vertically integrated with basic pathophysiology. In year three, focus is placed upon pathophysiology and therapeutics. In the spring semester of 2014, all lectures in the year three hematology block were didactically delivered in-class. Flipped classroom sessions were not conducted and no online discussion boards were used. Blended learning (BL) was introduced in the same module in the spring semester of 2015. Five out of twenty nine lectures were converted to PowerPoint presentations with instructor voice-overs. Microsoft PowerPoint 2007 was used with the ‘record slide show’ option and 13.5 kb/second stereo settings. These were then posted on the Alfaisal University learning management system, Moodle, and were subsequently not delivered in-class. Additionally, asynchronous discussion boards were created where students could interact with their peers and instructors regarding the online lectures and clarify concepts if required. A ‘Flipped Classroom’ was also arranged where a number of multiple choice questions and application exercises relating to the online and traditional lectures were placed.

Summative assessment results of the 2015 batch of students were compared with the previous batch as a historical control for any statistical significance. The proportion of students achieving grades A to C and grade F were compared for any improvement. Student performance of the class of 2015 was also compared between multiple choice questions from objectives being covered in online lectures versus traditional lectures.

### Data collection

Student feedback regarding their blended learning experience was collected through a self-developed questionnaire, comprising rating of seven items on a five point Likert scale, enquiring about their BL experience, objective understanding, online discussion board use, whether BL was useful for exam preparation, and its comparison to didactic teaching. Students were also asked their opinions whether the discussion board should be graded, and what proportion of lectures they would prefer to have as e-lectures rather than didactic. An open ended question about suggestions to improve the blended learning experience was also included. Questionnaires were distributed and collected during problem-based learning (PBL) sessions at the end of the module.

### Statistical analysis

Only descriptive statistics including mean of responses and distribution of students’ final grades in summative assessment were used. Student t-test was done to assess any statistical significance using SPSS version 21.

## Results

### Student evaluations

Approximately 22% of participants felt all lectures should be didactic compared to 73.1% of respondents who felt that some degree of online lectures would be preferable. 36% of responders felt it preferable for 20% of total lectures to be delivered online. The majority of students (69%) expressed satisfaction with BL as a novel, relevant, and effective learning approach. Almost 71% of students reported BL aided in exam preparation and clarification of objectives and concepts. Approximately 81% of students felt that BL was better compared to traditional didactic teaching approaches, while the same number of students thought that this online resource was relevant to their learning. Almost 42% of students remained neutral about their asynchronous discussion board experience. When asked about assigning grades to the discussion board, the majority of students (85%) disagreed, while 11.8% agreed and 3.2% did not answer.

Forty-one responses contained suggestions for BL improvement. Several students demonstrated preference for the use of blended learning in general, and requested inclusion of more topics via BL, such as “provide extra lectures through online”, “include in more blocks and courses as an integral learning course.” and “have all lectures or as many as possible available this way, give grades on Moodle, or assign students to talk about a topic and also place it on discussion board and grade it.”

Another group of students preferred the use of online lectures in basic concepts only. Their stated ideas included “only involve the topics that have been previously discussed and use blended learning only to tackle revision concepts, but didactic learning is needed for new material”, “Basic concepts that were already taught & are being taught in 3rd year should be covered that way”, and “keep the basics in online format only, We don't know how it would be for dense topics.”

Certain students identified the limited interaction with the instructor as a barrier to their learning experience. One of them stated “online lectures reduce the chance of me interacting and asking/discussing topics with the professor and fully understanding the topic”.                 

### Comparison of summative assessment

The mean grade between 2014 (155 students) and 2015 (154 students) (78.815 and 79.0584, respectively) did not significantly differ (t_(__307) _= 0.142, p=0.887). There were a slightly increased number of students passing with grades A and B in 2015 as compared to 2014. The number of students attaining grade C and F were also lower (but not statistically significant) in the 2015 batch compared to 2014.

An item-wise comparison of student performance on multiple choice questions in the summative assessment showed no statistically significant difference in student performance on items that came from didactic lectures versus online lectures (t_(76)_=1.02, p=0.311).

## Discussion

In recent times, the Ministry of Education in Saudi Arabia has taken numerous steps to integrate the use of technology in its educational system.^12^ E-learning has the potential to enhance quality of education and reach the ever expanding student population in the Kingdom.^12^ Very few studies have investigated the application of blended learning systems in educational institutions in the Middle East. The objective of our study was to compare conventional didactic modes of instruction to the fairly contemporary blended learning approach, using one particular block as an example. The current study focused on two main aspects:  student satisfaction, and academic performance. Our current results suggest significant student satisfaction with a BL approach. Analysis and comparison of grades show a slight, but not statistically significant, improvement in academic performance of students taught via BL, perhaps suggesting that BL enhances the educational experience, without impacting significantly upon summative outcomes. Indeed, with most examinations, students have to resort to more ‘traditional’ ways of revising.

We propose that increasingly replacing didactic with online lectures would significantly enhance student performance. Kiviniemi et al, demonstrated a mediocre improvement of outcomes in their study, albeit at the graduate and not undergraduate level.^2^ Our results suggest BL will presumably show similar enhancements in undergraduate medical education as well. A BL approach yielded academically superior results for evidence based medicine, with students preferring such an approach to didactic teaching.^13^ It is increasingly being accepted that methods such as BL are generally enjoyed by students, and increasing voices are suggesting its superiority over traditional didactic approaches.

BL methodology has the additional benefit of material being available online for review and re-visiting, unlike one-time class room sessions^2^^,^^14^ ; this was also supported by our survey. Our students had varying opinions toward the extent of blending that would be optimum. At the moment we started with approximately 17% of lectures being placed online as PowerPoint presentation with voice over for easy access. In the future this component could be increased gradually over the years for a more optimum blend.

### Student satisfaction survey

A consistently positive response was noted when students were questioned about the efficacy and versatility of BL, also acknowledging that using multiple sources of learning increased conceptual understanding and exam preparation. Students were, however, mostly ambivalent towards the use of the discussion board, with some asserting ignorance regarding its existence or failure to understand its use. When asked to choose between didactic teaching and BL, students held a mainly neutral stance, with some leaning towards BL. Collectively, however, students were open to experiencing innovative approaches to make their educational experience more productive and enjoyable, akin to those reported in other studies.^2^^, ^^5^ We speculate that online modalities of learning have an enormous scope in the future of education. Even students with initial concerns about such methods expressed enthusiasm towards it after having utilized them.^15^

### Flipped classroom

The second instructional BL strategy was the Flipped classroom model, a mode of education gaining increasing acceptance in the literature.16-18 A recent study indicated that the majority of students feel that the flipped classroom approach was better at fulfilling the learning objectives when compared to didactic teaching sessions,^16^^,^^17^ a trend noted in our student survey as well. Flipped classrooms improve student understanding, concept clarification through increased discussion time and engagement with faculty.^16^^,^^17^ While the ideal number of sessions to be deployed remains under investigation, however it is encouraging that active learning strategies such as flipped classrooms, team based learning, problem based learning, and case based learning are slowly replacing passive didactic methods like lectures. We would also like to emphasize that students need to come prepared to get the most benefit out of these sessions.^18^

### Asynchronous discussion boards

Another BL modality applied was the creation of asynchronous discussion boards. The concept of asynchronous discussion boards has several advantages as it provides students with a space to discuss and clarify concepts learnt in class and online.^19^ Like any new modality there are problems and challenges including management of sheer volume of students in a discussion board, developing acceptance among stakeholders, training of faculty and staff and engaging students to adopt and adapt. Our results indicate that students were not very enthusiastic to engage on the discussion boards. Posting online quizzes with students being required to answer on the discussion board may improve their participation. Also, most of the students did not want the discussion boards to be graded which needs to be taken into consideration while striving for student acceptance of new modalities.

### Limitations of the study

A major limitation of this study was that the BL approach was applied to one module only, limiting the scope and impact of this study. We were thus unable to determine which specific course components in a blended learning environment would be most beneficial to student success, as we examined the course and examination as a whole. In future a closer examination of approached coupled with exam component analyses from online versus didactic lectures may provide a clearer picture of modality effectiveness. Furthermore, broader implementation of BL in the curriculum would require training of more faculty members in such appraoches. Students and faculty may need training to access and navigate through e-learning resources. A large sample size would most likely help improve the statistical significance and power of studies like these. There are many elements within blended learning and each of them needs to be analyzed in order to create the most time and cost efficient educational system possible.

## Conclusions

We have reported one of the first applications of blended learning and flipped classroom in the Kingdom of Saudi Arabia. Our results indicate that these are promising modalities that enhance student satisfaction. They encourage independent learning and increased student engagement in class, thereby surpassing traditional lecture methods which have been criticized to encourage passivity among students. Online lectures allow students to pace themselves individually and eliminate the barriers of time and location. Flipped classrooms facilitate productive discussion and increased student interaction during valuable class time, enhancing their abilities to reason and apply learned information. Through the adoption of a blended method of instruction, learning experiences can be enriched at various levels of undergraduate and postgraduate education. We suggest that further research is needed to establish the academic outcomes of blended learning in order to create the most efficient instructional approach feasible.

### Acknowledgements

Authors would like to thank Dr. Junaid Kashir , Assistant professor, College of Medicine, Alfaisal University, Riyadh, Saudi Arabia, for critically reviewing this article and providing valuable feedback and Dr. Peter Cahusac, Associate Professor, College of Medicine, Alfaisal University, Riyadh, Saudi Arabia for performing statistical data analyses.

### Conflict of Interest

The authors declare that they have no conflict of interest.

## References

[1] Ruiz JG, Mintzer MJ, Leipzig RM (2006). The impact of E-learning in medical education.. Acad Med.

[2] Kiviniemi MT (2014). Effects of a blended learning approach on student outcomes in a graduate-level public health course.. BMC Med Educ.

[3] de Jong N, Savin-Baden M, Cunningham AM, Verstegen DM (2014). Blended learning in health education: three case studies.. Perspect Med Educ.

[4] Sancho P, Corral R, Rivas T, González MJ, Chordi A, Tejedor C (2006). A blended learning experience for teaching microbiology.. Am J Pharm Educ.

[5] Edginton A, Holbrook J (2010). A blended learning approach to teaching basic pharmacokinetics and the significance of face-to-face interaction.. Am J Pharm Educ.

[6] Jokinen P, Mikkonen I (2013). Teachers' experiences of teaching in a blended learning environment.. Nurse Educ Pract.

[7] Curran VR, Fleet LJ, Kirby F (2010). A comparative evaluation of the effect of Internet-based CME delivery format on satisfaction, knowledge and confidence.. BMC Med Educ.

[8] Rotellar C, Cain J (2016). Research, Perspectives, and Recommendations on Implementing the Flipped Classroom.. Am J Pharm Educ.

[9] Ojennus DD (2016). Assessment of learning gains in a flipped biochemistry classroom.. Biochem Mol Biol Educ.

[10] Liebert CA, Lin DT, Mazer LM, Bereknyei S, Lau JN (2016). Effectiveness of the Surgery Core Clerkship Flipped Classroom: a prospective cohort trial.. Am J Surg.

[11] Rowe M, Frantz J, Bozalek V (2012). The role of blended learning in the clinical education of healthcare students: a systematic review.. Med Teach.

[12] Alebaikan R, Troudi S (2010). Blended learning in Saudi universities: challenges and perspectives.. Research in Learning Technology.

[13] Ilic D, Hart W, Fiddes P, Misso M, Villanueva E (2013). Adopting a blended learning approach to teaching evidence based medicine: a mixed methods study.. BMC Med Educ.

[14] Delf P (2013). Designing effective eLearning for healthcare professionals.. Radiography.

[15] Abdelhai R, Yassin S, Ahmad MF, Fors UG (2012). An e-learning reproductive health module to support improved student learning and interaction: a prospective interventional study at a medical school in Egypt.. BMC Med Educ.

[16] Veeramani R, Madhugiri VS, Chand P (2015). Perception of MBBS students to "flipped class room" approach in neuroanatomy module.. Anat Cell Biol.

[17] McLaughlin JE, Roth MT, Glatt DM, Gharkholonarehe N, Davidson CA, Griffin LM, Esserman DA, Mumper RJ (2014). The flipped classroom: a course redesign to foster learning and engagement in a health professions school.. Acad Med.

[18] Pierce R, Fox J (2012). Vodcasts and active-learning exercises in a "flipped classroom" model of a renal pharmacotherapy module.. Am J Pharm Educ.

[19] Salmon G. E-moderating: The key to teaching and learning online. New York: Routledge; 2011.

